# Correlation between CT growth patterns and invasiveness progression in neoplastic subcentimeter sub-solid nodules

**DOI:** 10.1080/07853890.2025.2596471

**Published:** 2025-12-08

**Authors:** Ting Li, Can Ding, Shan-tong Yan, Xue-feng Jiang, Fa-jin Lv, Zhi-gang Chu

**Affiliations:** Department of Radiology, The First Affiliated Hospital of Chongqing Medical University, Chongqing, China

**Keywords:** Adenocarcinoma, computed tomography, follow-up studies, sub-solid nodules

## Abstract

**Background:**

Size and/or attenuation growth of neoplastic sub-solid nodules (SSNs) potentially indicate progression in invasiveness. This study aims to clarify the correlation between growth patterns based on size and attenuation and the invasiveness progression in subcentimeter neoplastic SSNs.

**Methods:**

From December 2018 to October 2024, 530 patients with 629 neoplastic SSNs (initial diameter <10 mm) were retrospectively enrolled. Based on CT changes (interval ≥3 months), the lesions were divided into four groups: I, no growth in size or attenuation; II, growth in both size and attenuation; III, growth only in size; and IV, growth only in attenuation. The relationship between growth patterns and pathological results was investigated, and the key CT indicators suggestive of invasiveness progression were determined.

**Results:**

Lesions in groups I, II, III, and IV were 424 (67.4%), 76 (12.1%), 84 (13.4%), and 45 (7.1%), respectively. The invasive adenocarcinomas (IACs) and invasive lesions (ILs) were more prevalent in groups II (38.2%, 78.9%) and III (11.9%, 52.4%) compared to group I (3.8%, 34.0%) (all *p* < 0.05). However, IACs (6.7%) and ILs (44.4%) in group IV were comparable to those in group I (all *p* > 0.05). The tumor mass was a more effective indicator for predicting the progression to IAC and ILs in group II (area under the curve [AUC] = 0.85, 0.76) and group III (AUC = 0.75, 0.65) (all *p* < 0.05).

**Conclusion:**

Regarding the subcentimeter SSNs, size growth, especially with concurrent attenuation increase, indicate a higher possibility of invasiveness progression, with mass being the primary predictor.

## Key Messages

1. Different growth patterns of subcentimeter neoplastic SSNs indicate varying degrees of invasiveness progression.
2. Growth only in attenuation does not indicate significant progression in invasiveness.
3. Size growth, especially with concurrent attenuation increasing, indicate a higher possibility of invasiveness progression.
4. Mass is the primary indicator of invasiveness progression in those with size growth.

## Introduction

The widespread adoption of low-dose computed tomography (LDCT) has significantly increased the detection of sub-solid nodules (SSNs) [1]. Persistent SSNs often represent lesions within the pulmonary adenocarcinoma spectrum [2,3]. Current guidelines recommend different surveillance strategies based on factors such as nodule size and the presence of solid component, given their indolent biological behavior and favorable prognosis [4–10]. Generally, neoplastic nodules with a diameter <10 mm exhibit relatively weak malignant behavior, with histology predominantly showing precursor lesions [11,12]. Therefore, subcentimeter neoplastic SSNs should be prioritized for long-term follow-up rather than aggressive management, and management strategies could be made by monitoring their changes.

During follow-up, neoplastic SSNs may remain stable or exhibit various growth changes in CT features, such as increases in diameter, attenuation, and solid components. Based on these changes, the progression of invasiveness (transformation from low invasiveness to high invasiveness) in lesions can be empirically evaluated [13,14], serving as a reference for subsequent management [4,6,7]. However, the growth patterns of SSNs can vary significantly, and the correlation between different growth patterns and the risk of invasiveness progression remains unclear. Therefore, to better manage subcentimeter neoplastic SSNs, it is necessary to identify the CT changes that are closely related to invasiveness progression.

Previous studies have revealed that SSNs with larger diameter, volume and a greater solid component are more likely to exhibit growth, with a higher probability of invasive lesions among the growing SSNs [9,15–19]. Additionally, lesions that progress to invasive forms typically grow faster [9,15]. The growth rate of SSNs correlates with various CT features [20], and there are positive correlations between accelerated growth and factors such as diameter, solid component size, and volume of SSNs [15–17]. The CT attenuation is helpful for evaluating invasiveness and growth risk but its predictive value remains controversial [5,21–23]. Furthermore, recent studies have confirmed that a combination of clinical, radiological, and radiomic features can better predict the invasiveness of SSNs [24,25]. While these studies have identified risk factors for SSN growth and the relationship between invasiveness and growth, to our knowledge, no study has categorized different SSN growth patterns, nor clarified whether specific growth patterns indicate invasiveness progression in neoplastic SSNs, particularly for subcentimeter ones.

We hypothesize that nodules exhibit different growth patterns during follow-up, and that not all patterns indicate invasiveness progression. Therefore, this study aims to clarify the correlation between various CT growth patterns (based on size and CT attenuation) and the progression of invasiveness in neoplastic subcentimeter SSNs. Furthermore, it seeks to determine which indicators are most effective for assessing invasive progression.

## Materials and methods

The study was conducted in accordance with the Declaration of Helsinki (as revised in 2013), and the study protocol was approved by the ethics committee of The First Affiliated Hospital of Chongqing Medical University (No. 2025-118-01). The requirement for written informed consent was waived due to the retrospective nature of the study. All the personal identification data were anonymized and de-identified before analysis.

### Patients’ selection

**Figure 1.**
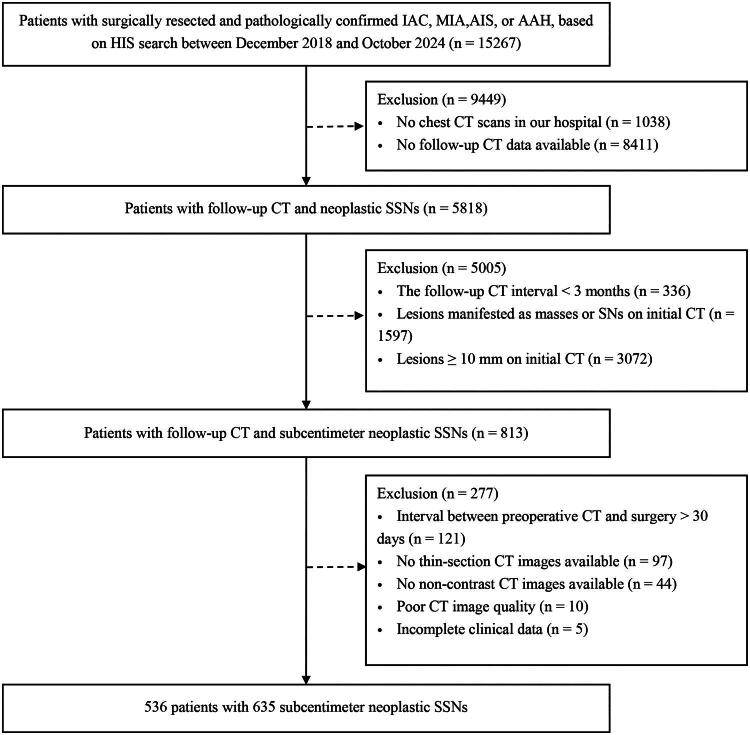
Flowchart of study population. IAC, invasive adenocarcinoma; MIA, minimally invasive adenocarcinoma; AIS, adenocarcinoma *in situ*; AAH, atypical adenomatous hyperplasia; HIS, hospital information system; CT, computed tomography; SSNs, subsolid nodules; SNs, solid nodules.

This study retrospectively enrolled patients with surgically resected pulmonary lesions at the First Affiliated Hospital of Chongqing Medical University from December 2018 to October 2024. Patients who underwent only biopsy were excluded due to the limited capacity of biopsy to adequately differentiate pathological subtypes. Pathology reports were reviewed through the Pathology Module of the Hospital Information System (HIS), and a total of 15,267 patients pathologically confirmed to have invasive adenocarcinoma (IAC), minimally invasive adenocarcinoma (MIA), adenocarcinoma *in situ* (AIS), or atypical adenomatous hyperplasia (AAH) following surgical resection were identified. Their chest CT images were retrieved through the Picture Archiving and Communication System (PACS) (version 3.1.S19.5, Carestream Vue, Carestream), and 1,038 cases were excluded due to the absence of the chest CT data in our hospital. Among the remaining 14,229 patients, 5,818 cases with follow-up CT data were identified. After excluding patients with a follow-up interval of < 3 months or cases with lesions that did not manifest as SSNs or SSNs ≥ 10 mm on the initial CT, 813 eligible patients were identified. SSNs comprise part-solid nodules (PSNs) and pure ground-glass nodules (pGGNs). PSNs are defined as nodules containing both ground-glass opacity and solid components, whereas pGGNs exhibit no solid components [7]. Subsequently, 121 patients with a preoperative CT to surgery interval greater than 30 days, 97 without thin-section CT data, 44 with only contrast-enhanced CT data, 10 with artifacts on CT images, and 5 without complete clinical data were excluded. Ultimately, this study included 635 SSNs from 536 patients. The patient selection procedure is shown in Figure 1.

### CT protocol

The chest CT scans were performed using one of the following CT scanners with similar scanning parameters and reconstruction algorithms: SOMATOM Perspective, SOMATOM Definition Flash, SOMATOM Force (Siemens Healthineers, Erlangen, Germany), Discovery CT750 HD (GE Healthcare, Milwaukee, WI, USA), and Aquilion ONE pureViSION (Canon Medical Systems, Japan). Scans were obtained with patients in full inspiration to minimize breathing artifacts. Changes in SSNs were evaluated based on the non-contrast CT images, which were acquired with the following settings: tube voltage of 100–110 kVp; tube current time of 40–140 mAs (with automatic tube current modulation); scanning slice thickness of 5 mm; rotation time of 0.5 s; pitch of 1–1.1; collimation of 0.6 or 0.625 mm; reconstruction slice thickness and interval of 0.625–1.5 mm; and matrix of 512 × 512. Images were obtained using mediastinal (width, 350–400 HU; level, 20–40 HU) and lung (width, 1,200–1,600 HU; level, −500 to −700 HU) window settings. All SSNs were evaluated on the lung window.

### Clinical data and image analysis

Patients’ clinical data were obtained using the electronic medical record system (Winning Health Technology, China). Clinical information included age, sex, follow-up period, smoking history, baseline diseases, personal history of malignancy, and family history of malignancy. CT data were independently reviewed by two radiologists (T.L. and C.D. with 5 and 3 years of experience in chest CT interpretation, respectively) on a PACS workstation (version 3.1.S19.5, Carestream Vue, Carestream). In cases of disagreement between the readers, a senior radiologist (Z.G.C. with 17 years of experience in chest CT interpretation) provided the final assessment.

The following features of SSNs were analyzed based on initial and preoperative CT images: (I) CT type (pGGN or PSN); (II) distribution (upper, middle, or lower lobes); (III) size (the average of the longest diameter and its perpendicular diameter in the same section); (IV) shape (regular or irregular); (V) boundary (well-defined or ill-defined); (VI) mean CT attenuation; (VII) size of the solid component in PSNs; (VIII) volume; (IX) mass; and (X) other morphological features (lobulation, spiculation, pleural indentation, vacuole sign, and air bronchogram). For PSNs, the measurement of nodule size was performed on the whole lesion including both the solid component and the peripheral ground-glass component. The measurement of the solid component in PSNs was performed on their largest section by averaging the longest diameter and its perpendicular diameter (Figure S1).

The measurement of CT attenuation of lesions was performed on their largest section on axial or multi-planar reconstruction (MPR) images containing the main component. CT attenuation of lesions was measured three times in the same section using a region-of-interest cursor to calculate the mean value, with the measurement area covering two-thirds of the lesion while avoiding vessels and bronchioles [26]. The volume and mass of lesions were measured using artificial intelligence software (InferRead CT Lung, InferVision Medical Health, China). Size growth of SSNs was defined as an increase in diameter of ≥ 1.5 mm or volume of ≥ 25% [3,6,27–29]. Attenuation growth was defined as an increase in mean CT value of ≥ 50 Hounsfield Units (HU), new solid component development in pGGN, or an increase in solid component size of ≥1.5 mm in PSN [7]. While no authoritative guidelines specify the threshold of mean CT attenuation increase that defines nodule growth, and thresholds used in previous studies were varied [3,30], a threshold of 50 HU was set in this study based on the following reasons: (1) this study specifically focused on subcentimeter SSNs, which predominantly presented as pGGNs and had lower CT attenuation; (2) those nodules with visible attenuation growth typically had an increase in CT value of ≥50 HU. Based on growth patterns during follow-up, the lesions were divided into four groups: I (no growth in size or attenuation), II (growth in both size and attenuation), III (growth only in size), and IV (growth only in attenuation).

### Pathological analysis

AAH and AIS are classified as precursor glandular lesions. AAH typically presents as focal atypical alveolar epithelial hyperplasia, while AIS is a lepidic-pattern lesion measuring ≤3 cm without stromal, vascular, or pleural invasion. MIA and IAC are categorized as adenocarcinomas. MIA is characterized by a predominantly lepidic growth pattern with an invasive focus ≤0.5 cm in greatest dimension, whereas IAC exhibits definitive invasive growth with various histologic patterns (Figure S2) [13,31]. The pathological slides of patients in group IV were reviewed by a pathologist. During the review, the histological components that may cause increased CT attenuation were analyzed, including tumor cells, alveolar collapse, fibrous tissue proliferation, and others. Throughout the entire process, the pathologist was blinded to the primary pathological diagnosis of the patients.

### Statistical analysis

Statistical analyses were performed using SPSS software (Version 27; IBM, Armonk, NY, USA), R language (RStudio 2024.12.1; Posit Software, PBC), and GraphPad Prism software (Version 9.5.0; GraphPad Software, San Diego, CA, USA). The Shapiro–Wilk test was used to assess the normality of continuous variables. Normally distributed data were reported as mean ± SD, non-normally distributed data as median (IQR), and categorical data as *n* (%). Univariate analysis was conducted using Pearson’s χ^2^ test, Fisher’s exact test, Mann–Whitney *U* test, or Kruskal–Wallis test. Assessment performance was evaluated by ROC analysis, with area under the curve (AUC) comparisons performed using the DeLong test. Multiple comparisons were adjusted using the Bonferroni correction, and the statistical significance level was set at α = 0.05/*n* (where *n* is the number of comparisons). Variables with statistical difference in univariate analysis were further included in multivariate logistic regression analysis. Firth’s penalized logistic regression was employed for multivariate analysis in subgroups. Before constructing the multivariate model, multicollinearity for the variables was assessed by calculating the variance inflation factor (VIF) and tolerance. A VIF of >5 and a tolerance of <0.2 were considered indicative of collinearity. Inter-reader agreement was assessed by intraclass correlation coefficient (ICC) for nodule size, CT value, and solid component size assessment, and Cohen’s kappa for nodule type [32]. A *p* value <0.05 was considered statistically significant.

## Results

### Inter-reader agreement of CT features

Table S1 summarizes the inter-reader agreement for the CT features of the lesions. For continuous variables, nodule size, CT value and solid component size were excellent (0.93–0.96). For categorical indicators, the agreement for CT pattern was substantial (0.80), while that for other morphological features was almost perfect (0.81–0.91).

### Patients’ clinical characteristics

Among the 635 SSNs, 2 pGGNs and 4 PSNs progressed to SNs, which were confirmed as IACs. The remaining 629 SSNs in 530 patients were further studied, which included 424 (67.4%) in group I, 76 (12.1%) in group II, 84 (13.4%) in group III, and 45 (7.1%) in group IV. Single and multiple (≥2) lesions were observed in 455 (85.8%) and 75 (14.2%) patients, respectively. The mean follow-up period for the 530 patients was 473 (214–901) days.

The patients’ clinical characteristics are presented in Table 1. Smokers were more common in group II (*p* < 0.05), while those in the other three groups were similar (*p* > 0.05). Males and individuals with a history of malignancy were more common in group II than in group I (*p* < 0.05). Compared to patients in groups I and IV, those in groups II and III were older, and their follow-up intervals were longer (all *p* < 0.05).

**Table 1.** Patients’ clinical characteristics.

Characteristics	Group I (*n* = 336)		Group II (*n* = 69)		Group III (*n* = 81)		Group IV (*n* = 44)		*p* value
Sex									0.035^a^
Male	80 (23.8)		28 (40.6)		24 (29.6)		11 (25.0)		
Female	256 (76.2)		41 (59.4)		57 (70.4)		33 (75.0)		
Age (years)^#^	53 (45–60)		60 (53–68)		58 (52–63)		55 (41–60)		<0.001^c^
Follow-up period (days)^#^	338 (163–651)		1064 (739–1481)		931 (602–1305)		373 (214–816)		<0.001^c^
Smoking history									<0.001^a^
Smoker	38 (11.3)		55 (79.7)		12(14.8)		5 (11.4)		
Non-smoker	298 (88.7)		14 (20.3)		69 (85.2)		39 (88.6)		
Baseline disease									
Diabetes	15 (4.5)		8 (11.6)		5 (6.2)		1 (2.3)		0.108^b^
Hypertension	54 (16.1)		20 (29.0)		17 (21.0)		9 (20.5)		0.083^a^
None	274 (81.5)		47 (68.1)		60 (74.1)		35 (79.5)		0.065^a^
Personal history of malignancy	71 (21.1)		26 (37.7)		26 (32.1)		9 (20.5)		0.01^a^
Family history of malignancy	57 (17.0)		10 (14.5)		15 (18.5)		9 (20.5)		0.851^a^

Note: Unless otherwise stated, data presented as the number of participants with the percentage in parentheses.

^#^ Median (interquartile range [IQR]).

^a^ Calculated by Pearson’s chi-square test.

^b^ Calculated by Fisher’s exact test.

^c^ Calculated by the Kruskal–Wallis *H* test.

### Comparison of pathological results across groups

**Figure 2.**
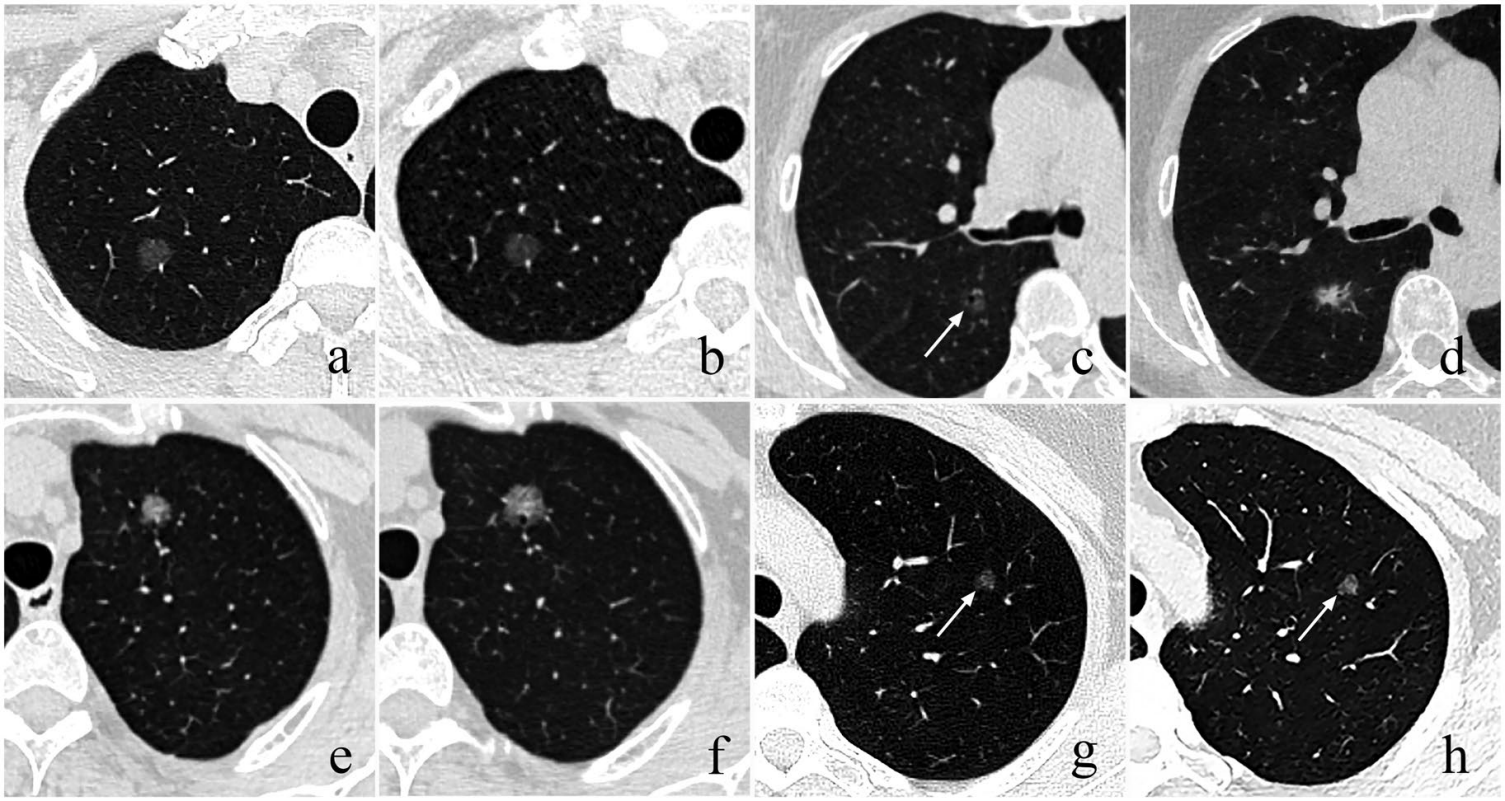
SSNs with different growth patterns. (a, b) A 45-year-old female has a pGGN (diameter: 9.2 mm; volume: 283 mm³; CT value: −690 HU) located in the RUL. On follow-up CT (interval: 36.4 months), it shows no significant growth (diameter: 9.2 mm; volume: 318 mm³; CT value: −684 HU). After operation, it is confirmed as AIS. (c, d) A 75-year-old female has a pGGN (white arrow) (diameter, 8.9 mm; volume, 188 mm³; mass, 82 mg; CT value, −699 HU) located in the RLL. On follow-up CT (interval: 65.6 months), it shows diameter growth of 6.9 mm, volume growth of 1336 mm³, mass growth of 923 mg, and a new 7.2 mm solid component. After operation, it is confirmed as IAC. (e, f) A 58-year-old female has a pGGN (diameter, 9.5 mm; volume,261 mm³; mass, 172 mg; CT value, −451 HU) in the LUL. On follow-up CT (interval: 15.1 months), it shows diameter growth of 2.3 mm, volume growth of 409 mm³, and mass growth of 191 mg, but no significant growth in CT value (−442 HU). After operation, it is confirmed as MIA. (g, h) A 58-year-old female has a pGGN (white arrow) (diameter, 6.7 mm; volume, 104 mm³; CT value, −697 HU) in the LUL. On follow-up CT (interval: 43.4 months), it shows growth in CT value (−637 HU), but no significant growth in diameter (6.7 mm) and volume (107 mm³). After operation, it is confirmed as AIS. SSNs, subsolid nodules; pGGN, pure ground-glass nodule; CT, computed tomography; HU, Hounsfield unit; AIS, adenocarcinoma *in situ*; MIA, minimally invasive adenocarcinoma; IAC, invasive adenocarcinoma; RUL, right upper lobe; LUL, left upper lobe; RLL, right lower lobe.

Among 629 SSNs, 58 (9.2%), 210 (33.4%), 340 (54.1%), and 21 (3.3%) were finally confirmed as IACs, MIAs, AISs, and AAHs, respectively. Table 2 presents the pathological results of lesions in different groups. Compared with group I (IACs: 3.8%; invasive lesions [ILs] (MIA + IAC): 34.0%), IACs and ILs were more common in group II (IACs: 38.2%; ILs: 78.9%) and group III (IACs: 11.9%; ILs: 52.4%) (all *p* < 0.05), while no significant differences were observed in group IV (IACs: 6.7%; ILs: 44.4%) (*p* > 0.05). Representative CT images for each group are presented in Figure 2.

**Table 2.** Pathological results of lesions in different groups.

	Group I (*n* = 424)	Group II (*n* = 76)	Group III (*n* = 84)	Group IV (*n* = 45)	*p* value
AAH	17 (4.0)	0 (0.0)	2 (2.4)	2 (4.5)	0.105^b^
AIS	263 (62.0)	16 (21.1)	38 (45.2)	23 (51.1)	<0.001^a^
MIA	128 (30.2)	31 (40.8)	34 (40.5)	17 (37.8)	0.106^a^
IAC	16 (3.8)	29 (38.2)	10 (11.9)	3 (6.7)	<0.001^a^
ILs (MIA + IAC)	144 (34.0)	60 (78.9)	44 (52.4)	20 (44.4)	<0.001^a^

Note: Data are expressed as *n* (%).

AAH, atypical adenomatous hyperplasia; AIS, adenocarcinoma *in situ*; MIA, minimally invasive adenocarcinoma; IAC, invasive adenocarcinoma; ILs, invasive lesions.

^a^ Calculated by Pearson’s chi-square test.

^b^ Calculated by Fisher’s exact test.

### Multivariable analysis for predicting IAC and ILs based on clinical characteristics and growth patterns

The multivariable analysis for predicting IAC and ILs based on clinical characteristics and growth patterns are presented in Table 3. Progressive change in group II nodules was a significant indicator for predicting both IAC (odds ratio [OR] = 10.78, 95% confidence interval [CI]: 5.27–22.03; *p* < 0.001) and ILs (OR = 6.33, 95% CI: 3.46–11.57; *p* < 0.001). Similarly, progressive change in group III nodules was a significant indicator for predicting both IAC (OR = 2.50, 95% CI: 1.07–5.86; *p* = 0.035) and ILs (OR = 1.94, 95% CI: 1.19–3.15; *p* = 0.007). Additionally, older age was independently associated with a higher risk of IAC (OR = 1.05, 95% CI: 1.02–1.09; *p* = 0.001).

**Table 3.** Multivariable analysis for predicting IACs and ILs based on clinical characteristics and growth patterns.

Variables	IACs		ILs	
	*p* value	OR (95% CI)	*p* value	OR (95% CI)
Sex	0.163	1.764 (0.794–3.919)	0.815	0.944 (0.582–1.530)
Age	0.001	1.054 (1.021–1.087)	0.182	1.011 (0.995–1.027)
Smoking history	0.650	1.252 (0.475–3.289)	0.123	1.618 (0.877–2.982)
Personal history of malignancy	0.263	1.453 (0.756–2.792)	0.182	1.312 (0.881–1.955)
Group 1	**–**	**–**	**–**	**–**
Group 2	<0.001	10.777 (5.272–22.028)	<0.001	6.328 (3.462–11.566)
Group 3	0.035	2.499 (1.066–5.858)	0.007	1.939 (1.194–3.149)
Group 4	0.334	1.891 (0.520–6.883)	0.149	1.587 (0.848–2.972)

Note: IACs, invasive adenocarcinomas; ILs, invasive lesions; OR, Odds ratio; CI, Confidence interval.

Multivariate analysis adjusted for male sex, age, smoking history, personal history of malignancy, with group 1 as the reference.

### CT features of SSNs on initial and preoperative CT images

The CT features of SSNs on initial CT images are presented in Table 4. Irregular nodules were more common in groups II (11.8%) and III (10.7%) than in group I (3.5%) (all *p* < 0.05). Ill-defined nodules were more prevalent in groups II (26.3%) and IV (28.9%) than those in group I (10.1%) (all *p* < 0.05). A greater proportion of nodules with lobulation was observed in group II (18.4%) compared to group I (7.3%) (*p* < 0.05).

**Table 4.** Features of SSNs on initial CT scan.

Characteristics	Group I (*n* = 424)	Group II (*n* = 76)	Group III (*n* = 84)	Group IV (*n* = 45)	*p* value
Diameter (mm)^#^	6.9 (6.0–8.0)	6.8 (5.4–8.7)	6.7 (5.6–8.1)	7.5 (6.5–8.3)	0.271^c^
Attenuation (HU)^#^	−621 (−698, −530)	−600 (−683, −490)	−633 (−699, −527)	−597 (−673, −511)	0.273^c^
Volume (mm^3^)^#^	133 (93–201)	102 (64–221)	133 (74–183)	171 (104–224)	0.055^c^
Mass (mg)^#^	69 (44–103)	70 (34–126)	65 (38–102)	86 (53–115)	0.456^c^
Distribution					0.691^a^
Upper lobe	278 (65.6)	54 (71.1)	58 (69.0)	32 (71.1)	
Middle and lower lobes	146 (34.4)	22 (28.9)	26 (31.0)	13 (28.9)	
CT pattern					0.057^a^
pGGN	385 (91.3)	62 (81.6)	72 (85.7)	40 (88.9)	
PSN	37 (8.7)	14 (18.4)	12 (14.3)	5 (11.1)	
Solid component size (mm)^#^	3.1 (2.6–3.4)	3.9 (2.9–4.7)	3.0 (2.6–5.3)	2.7 (2.5–3.6)	0.209^a^
Shape					<0.001^b^
Regular	409 (96.5)	67 (88.2)	75 (89.3)	43 (95.6)	
Irregular	15 (3.5)	9 (11.8)	9 (10.7)	2 (4.4)	
Boundary					<0.001^a^
Ill-defined	43 (10.1)	20 (26.3)	13 (15.5)	13 (28.9)	
Well-defined	381 (89.9)	56 (73.7)	71 (84.5)	32 (71.1)	
Lobulation	31 (7.3)	14 (18.4)	12 (14.3)	5 (11.1)	0.011^a^
Spiculation	5 (1.2)	3 (3.9)	1 (1.2)	0 (0.0)	0.261^b^
Pleural indentation sign	19 (4.5)	6 (7.9)	4 (4.8)	3 (6.7)	0.515^b^
Vacuole sign	26 (6.1)	11 (14.5)	9 (10.7)	3 (6.7)	0.057^a^
Air bronchogram	27 (6.4)	9 (11.8)	10 (11.9)	3 (6.7)	0.173^a^

Note: Unless otherwise stated, data presented as the number of participants with the percentage in parentheses. CT, computed tomography; HU, Hounsfield units; pGGN, pure ground-glass nodule; PSN, part-solid nodule.

^#^ Median (interquartile range [IQR]).

^a^ Calculated by Pearson’s chi-square test.

^b^ Calculated by Fisher’s exact test.

^c^ Calculated by the Kruskal–Wallis *H* test.

The CT features of SSNs on preoperative CT images are presented in Table 5. Compared to the lesions on initial CT, the proportions of PSN (60.5% vs. 18.4%, *p* < 0.001) and lesions with irregular shape (35.5% vs. 11.8%, *p* < 0.001), well-defined boundary (92.1% vs. 73.7%, *p* = 0.003), lobulation (57.9% vs. 18.4%, *p* < 0.001), spiculation (13.2% vs. 3.9%, *p* = 0.042), pleural indentation (27.6% vs. 7.9%, *p* = 0.001), and air bronchogram (31.6% vs. 11.8%, *p* = 0.003) significantly increased in group II, and the proportion of lesions with lobulation (36.9% vs. 14.3%, *p* = 0.002) significantly increased in group III, while no significant change was observed in group I and IV.

**Table 5.** Features of SSNs on preoperative CT scan.

Characteristics	Group I (*n* = 424)	Group II (*n* = 76)	Group III (*n* = 84)	Group IV (*n* = 45)	*p* value
Diameter (mm)^#^	7.3 (6.2–8.4)	10.2 (8.2–11.9)	9.4 (8.1–10.6)	7.7 (6.6–8.5)	<0.001^c^
Attenuation (HU)^#^	−617 (−689, −527)	−485 (−589, −345)	−614 (−701, −512)	−524 (−628, −437)	<0.001^c^
Volume (mm^3^)^#^	145 (99–216)	340 (227–1129)	284 (180–434)	183 (107–243)	<0.001^c^
Mass (mg)^#^	73 (47–113)	218 (128–628)	137 (89–209)	90 (59–133)	<0.001^c^
CT pattern					<0.001^a^
pGGN	387(91.3)	30 (39.5)	72 (85.7)	38 (84.4)	
PSN	37 (8.7)	46 (60.5)	12 (14.3)	7 (15.6)	
Solid component size (mm)^#^	3.2 (2.7–3.6)	5.4 (3.9–7.2)	3.9 (2.8–5.6)	3.4 (3.0–4.0)	<0.001^c^
Shape					<0.001^a^
Regular	409 (96.5)	49 (64.5)	71 (84.5)	43 (95.6)	
Irregular	15 (3.5)	27 (35.5)	13 (15.5)	2 (4.4)	
Boundary					0.013^a^
Ill-defined	30 (7.1)	6 (7.9)	4 (4.8)	9 (20.0)	
Well-defined	394 (92.9)	70 (92.1)	80 (95.2)	36 (80.0)	
Lobulation	31 (7.3)	44 (57.9)	31 (36.9)	5 (11.1)	<0.001^a^
Spiculation	7 (1.7)	10 (13.2)	5 (6.0)	2 (4.4)	<0.001^b^
Pleural indentation	19 (4.5)	21 (27.6)	6 (7.1)	3 (6.7)	<0.001^a^
Vacuole sign	27(6.4)	19 (25.0)	10 (11.9)	3 (6.7)	<0.001^a^
Air bronchogram	27 (6.4)	24 (31.6)	12 (14.3)	3 (6.7)	<0.001^a^

Note: Unless otherwise stated, data presented as the number of participants with the percentage in parentheses.

CT, computed tomography; HU, Hounsfield units; pGGN, pure ground-glass nodule; PSN, part-solid nodule.

^#^ Median (interquartile range [IQR]).

^a^ Calculated by Pearson’s chi-square test.

^b^ Calculated by Fisher’s exact test.

^c^ Calculated by the Kruskal–Wallis *H* test.

### CT indicators for evaluating progression of invasiveness in groups II and III

**Figure 3.**
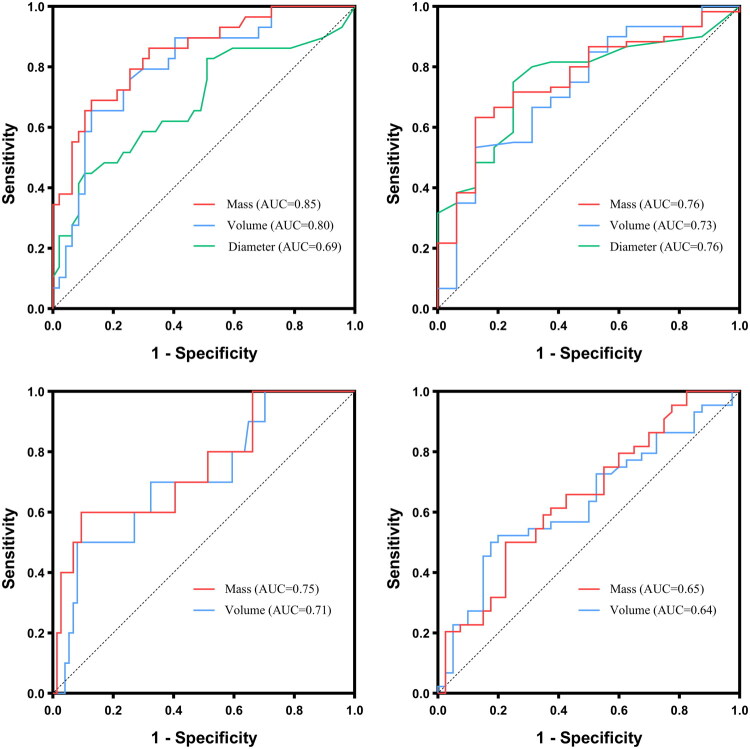
Receiver operating characteristic (ROC) curves of different indicators for discriminating IACs from non-IACs and ILs from non-ILs in groups II (a, b) and III (c, d).

Table 6 shows the efficiencies of different indicators for predicting IACs and ILs in groups II and III. In group II, mass exhibited significantly better performance than diameter and volume in predicting IACs (AUC: 0.85 vs. 0.80 and 0.69; all *p* < 0.05); and mass and diameter showed slightly better performance than volume for identifying ILs (AUC: 0.76 and 0.76 vs. 0.73) but without significant differences (all *p* > 0.05). In group III, mass demonstrated a slightly better performance than volume in identifying IACs (AUC: 0.75 vs. 0.71) and ILs (AUC: 0.65 vs. 0.64), but there were no significant differences (all *p* > 0.05) (Figure 3). To mitigate multicollinearity, only mass was selected for multivariable analysis because mass and volume exhibited multicollinearity in this study. The multivariate logistic regression analysis identified mass as an independent risk factor for predicting IAC in both groups II and III (group II: *p* = 0.031, OR = 1.004, 95% CI: 1.000–1.008; group III: *p* = 0.002, OR = 1.005, 95% CI: 1.002–1.011). However, mass was not an independent risk factor for predicting ILs (group II: *p* = 0.592; group III: *p* = 0.232) (Tables S2 and S3).

**Table 6.** Evaluation of different growth indicators for assessing IACs and ILs.

Group	Indicator	Cutoff	AUC*	*p* value	Sensitivity	Specificity
Group 2	Attenuation (HU)	90	0.61 (0.47, 0.74)	0.114	0.65	0.55
	Diameter (mm)	4.5	0.69 (0.56, 0.82)	0.007	0.45	0.89
	Volume (mm^3^)	395	0.80 (0.70, 0.90)	<0.001	0.66	0.87
	Mass (mg)	272	0.85 (0.76, 0.94)	<0.001	0.69	0.87
Group 2^#^	Attenuation (HU)	79	0.56 (0.40, 0.72)	0.437	0.68	0.50
	Diameter (mm)	2.1	0.76 (0.64, 0.88)	0.002	0.82	0.69
	Volume (mm^3^)	247	0.73 (0.59, 0.87)	0.005	0.53	0.88
	Mass (mg)	141	0.76 (0.64, 0.88)	0.001	0.63	0.88
Group 3	Diameter (mm)	2.0	0.63 (0.47, 0.80)	0.171	0.80	0.49
	Volume (mm^3^)	427	0.71 (0.55, 0.88)	0.029	0.50	0.92
	Mass (mg)	164	0.75 (0.58, 0.92)	0.01	0.60	0.91
Group 3^#^	Diameter (mm)	2.4	0.53 (0.41, 0.66)	0.588	0.46	0.68
	Volume (mm^3^)	186	0.64 (0.52, 0.75)	0.033	0.50	0.83
	Mass (mg)	91	0.65 (0.53, 0.77)	0.019	0.50	0.78

Note: AUC, area under the receiver operating characteristic curve; HU, Hounsfield units.

* Data in parentheses are 95% confidence intervals.

^#^ The study subjects were ILs.

### Pathological findings of SSN in group IV

**Figure 4.**
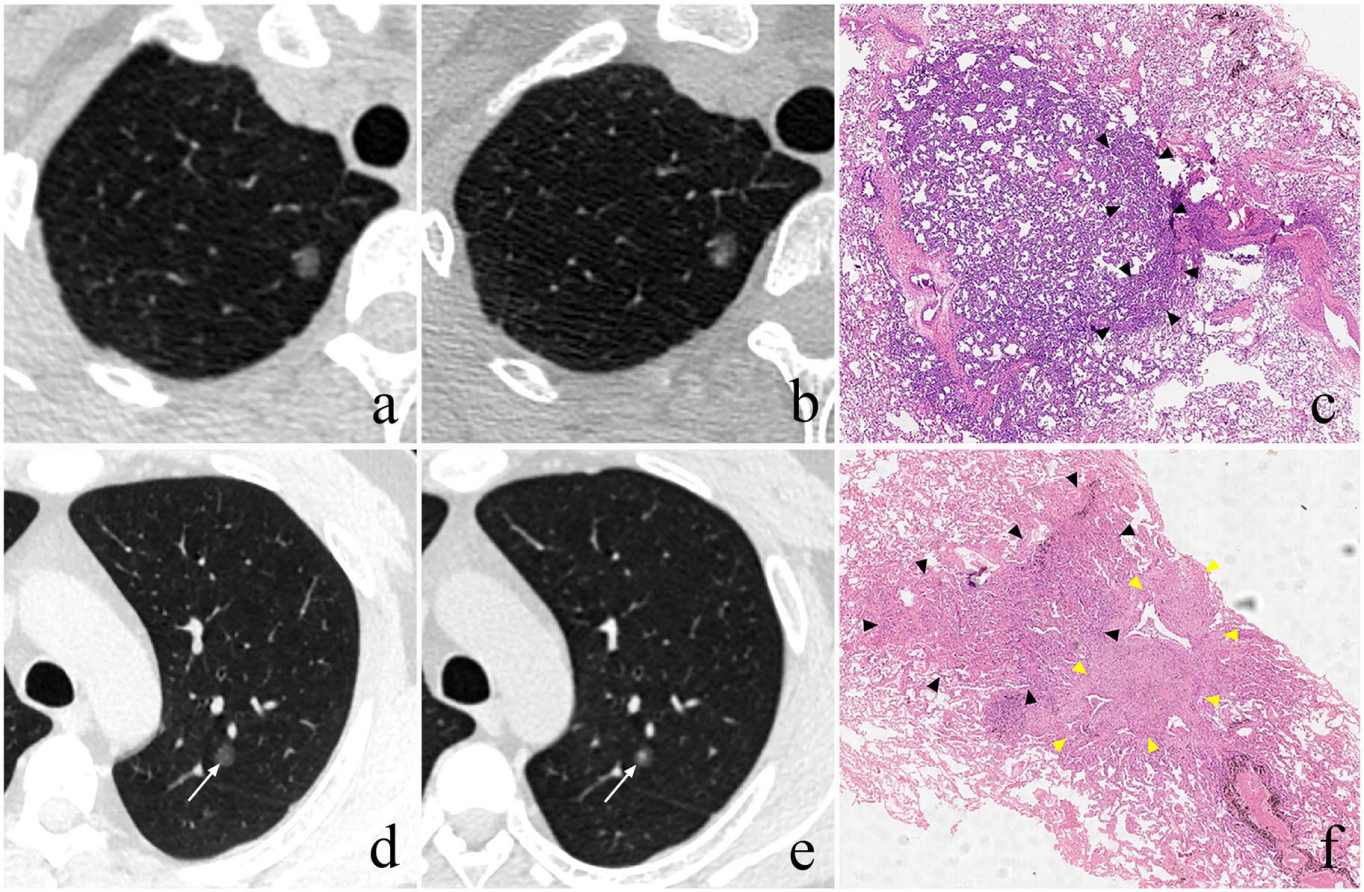
Representative cases demonstrating pathological characteristics of SSNs with attenuation growth only. (a–c) A 40-year-old female has a pGGN (diameter, 7.3 mm; volume, 171 mm³; CT value, −587 HU) located in the RUL. On follow-up CT (interval: 9.3 months), it shows an increase in CT value to −534 HU, but no significant growth in diameter (7.4 mm) and volume (184 mm³). After operation, it is confirmed as AIS, accompanied by alveolar collapse (black arrowheads). (d–f) A 38-year-old male has a pGGN (diameter, 7.2 mm; volume, 176 mm³; CT value, −696 HU) located in the LUL (white arrow). On follow-up CT (interval: 34.3 months), it shows CT attenuation increasing to −643 HU, but no significant growth in diameter (7.2 mm) and volume (179 mm³). After operation, it is confirmed as AIS, accompanied by alveolar collapse (black arrowheads) and fibrous tissue proliferation (yellow arrowheads). SSNs, subsolid nodules; pGGN, pure ground-glass nodule; CT, computed tomography; HU, Hounsfield unit; RUL, right upper lobe; LUL, left upper lobe; AIS, adenocarcinoma *in situ.*

Pathological analysis was performed on the 45 nodules in group IV, including 3 IACs, 17 MIAs, 23 AISs and 2 AAHs. Each SSN exhibited one or a combination of multiple non-neoplastic histological features, including alveolar collapse (21, 29.2%), inflammatory cell infiltration (21, 29.2%), fibrous tissue proliferation (16, 22.2%), and significant interstitial thickening (14, 19.4%) (Figure 4).

## Discussion

Subcentimeter SSNs are generally recommended for surveillance, with further management determined by changes observed during follow-up. Although subcentimeter SSNs display various growth patterns on CT images, not all patterns indicate invasiveness progression. The present study found that when lesions show growth solely in attenuation, this suggests a low likelihood of invasiveness progression. Conversely, when lesions increase in size, particularly accompanied by concurrent growth in attenuation, this indicates a higher likelihood of invasiveness progression. For lesions exhibiting growth patterns that suggest a progression in invasiveness, mass is the preferred indicator for further assessing their invasiveness.

Several studies reported that CT attenuation can be used for assessing the invasiveness of neoplastic SSNs [5,21,33–35], but its predictive value remains controversial [22,23]. In the present study, compared to non-growing subcentimeter SSNs, those that only presented growth in attenuation did not demonstrate a significant increase in invasiveness. The primary reason responsible for this finding may be that the attenuation growth in SSNs may result from either invasive progression or benign causes like alveolar collapse, inflammatory cell infiltration, interstitial thickening, fibrous tissue proliferation, and others [36,37]. These benign conditions can reduce air content in the lesion or increase tissue density, leading to an increase in attenuation. Therefore, the subcentimeter SSNs with only attenuation growth are more likely associated with those benign factors. The pathological analysis also confirmed that the non-invasive components accounting for the attenuation increase were highly prevalent in group IV. For subcentimeter SSNs that only exhibit an increase in attenuation, management can remain conservative without shortening the follow-up interval. However, when the subcentimeter SSNs transform into SNs, it should prompt diagnostic intervention, as this change strongly suggests they have progressed to IAC, warranting distinct clinical consideration.

In contrast, the current results indicate that for subcentimeter SSNs, CT follow-up should concentrate on monitoring any increase in size. Additionally, if there is a concurrent increase in attenuation, the likelihood of invasiveness progression is further elevated. These findings are consistent with the previous results that there was close relationship between nodule size and invasiveness in neoplastic SSNs [38–41]. Reported cutoff values of diameter for distinguishing MIA from AIS/AAH in SSNs, IAC from AAH/AIS/MIA in pGGNs and SSNs, and IAC/MIA from AAH/AIS in pGGNs and SSNs are 10.05 mm, 10.09 mm, 14.4, 10, and 14 mm, respectively [41–43]. Additionally, during the progression from AAH to IAC, nodules usually display an increase in size, which is accompanied by EGFR mutation and the evolution of the immune microenvironment toward a more suppressed state [44]. Therefore, in comparison to sole attenuation increasing, growth in size truly indicates an increase in invasiveness for subcentimeter SSNs, and an increase in attenuation based on an increase in size is more likely to represent the development of invasive components, warranting consideration of a shortened follow-up interval or diagnostic intervention.

For subcentimeter SSNs showing size growth during follow-up, appropriate indicators are required to better evaluate their invasiveness. Previous research has supported the potential of volume doubling time (VDT) in assessing substantial SSN growth and invasiveness progression [45]. Various studies have found that as an indicator integrating nodule size and attenuation, mass is recognized as a sensitive, stable, and accurate indicator for assessing SSN growth and invasiveness [14,38,39]. de Hoop et al. [46]. confirmed that mass as a more sensitive metric than diameter or volume for early detection of SSN progression. Qi et al. [39]. found that SSN mass showed superior performance in differentiating IAC from non-IAC compared to diameter or volume. Xiong et al. [38]. demonstrated that mass showed superior performance over diameter and CT morphological features in evaluating the invasiveness of SSNs. Additionally, Kim et al. [47]. reported that pGGN mass exhibited comparable capability to volume and diameter in differentiating IAC from non-IAC. Similarly, the present study revealed that the mass exhibits higher or comparable predictive value for assessing invasiveness progression in SSNs. Mass as an independent indicator demonstrated higher predictive efficacy in evaluating the progression to IAC. However, it was not effective for predicting ILs. The reason may be that the increase in mass from glandular precursor lesions to MIA is not significant compared to that from glandular precursor lesions to IAC during follow-up. Considering some SSNs may show little changes in diameter or attenuation during follow-up, mass as a comprehensive indicator may offer superior accuracy. Therefore, for nodules in groups II and III, measuring their mass is beneficial for adjusting the subsequent follow-up strategy.

Multiple studies have shown that morphological features, such as lobulation, spiculation, pleural indentation, vacuole sign, and air bronchogram, correlate with neoplastic SSN growth and invasiveness, typically indicating more advanced malignant progression [14,18,48,49]. Comparing the changes in CT features of the lesions between the initial and preoperative CT scans, the increase in features was most pronounced in group II, followed by group III, while groups I and IV showed little change. These findings are consistent with the correlation between growth patterns and invasiveness progression. Thus, these CT features may assist in evaluating the progression in invasiveness of neoplastic SSNs. However, their sensitivity and specificity are generally inferior to quantitative indicators like diameter, volume, and mass [38]. This may be because they were less common in SSNs than in SNs, especially in the smaller ones.

This study has several limitations. First, as this was a retrospective study, the follow-up intervals for nodules varied in different groups, however, since our primary focus was on nodule changes, these variations did not affect our results. Second, due to the use of different CT scanners during follow-up in some patients, there may be variability in CT attenuation measurements. Therefore, we established a reasonable attenuation growth threshold by integrating visual assessment and manual measurement while considering the attenuation characteristics of subcentimeter SSNs. Third, we assessed the invasiveness progression of lesions in different groups by comparing with nodules that show no significant growth; thus the differences in baseline characteristics might influence invasiveness assessment. In this study, there was no significant differences in key invasiveness-related parameters (e.g. diameter, attenuation, volume, mass, and CT type), and some different CT features had uncertain value for assessing invasiveness. Fourth, since this study focused on subcentimeter SSNs, the findings may not be suitable for larger SSNs. Additionally, as this was a single-center study, the sample size of the growth groups is relatively limited, especially for group IV, may constrain the generalizability of our findings. Multicenter, large-scale prospective studies are warranted to validate these results in the future.

## Conclusions

During follow-up, changes in size should be prioritized when monitoring subcentimeter SSNs. The subcentimeter SSNs exhibiting size growth, particularly with concurrent increases in attenuation, indicate a higher likelihood of invasiveness progression, which requires closer follow-up or diagnostic intervention. In contrast, nodules that show an increase in attenuation alone do not suggest significant progression in invasiveness, and continued routine follow-up may be firstly considered. However, if an SSN progresses to a solid nodule, surgical resection should be considered.

## Supplementary Material

Supplemental Material

## Data Availability

Upon reasonable request, the corresponding author can provide data to support the findings of this study.

## Clinical importance

Regarding the subcentimeter neoplastic SSNs, those showing growth in size, especially when accompanied by an increase in attenuation, highly indicate progression in invasiveness and should be closely monitored, rather than those with growth only in attenuation.
