# Supplementary material for: Correlation between CT growth patterns and invasiveness progression in neoplastic subcentimeter sub-solid nodules
Source: Ann Med. 2025 Dec 8;57(1):2596471. doi: 10.1080/07853890.2025.2596471 (PMC12687892; doi:10.1080/07853890.2025.2596471)
Supplement: Supplemental Material [file IANN_A_2596471_SM0678.zip › suppl_data/Clean copy - Supplementary Table - IANN-2025-4221.R1.docx]

**Table S1.** Inter-reader agreement of CT features

| Parameters | Metric^*^ | 95% CI |
| --- | --- | --- |
| Size | 0.94 | 0.92**–**0.96 |
| CT value (HU) | 0.93 | 0.87**–**0.96 |
| Solid component size | 0.96 | 0.95**–**0.97 |
| CT pattern | 0.80 | 0.75**–**0.85 |
| Shape | 0.81 | 0.75**–**0.86 |
| Boundary | 0.84 | 0.79**–**0.88 |
| Lobulation | 0.84 | 0.81**–**0.88 |
| Spiculation | 0.91 | 0.84**–**0.98 |
| Pleural indentation | 0.88 | 0.84**–**0.92 |
| Vacuole sign | 0.90 | 0.86**–**0.93 |
| Air bronchogram | 0.88 | 0.83**–**0.92 |

Note: ^*^ Metric represents ICC for continuous variables and kappa coefficient for categorical variables. Inter-reader agreement, as indicated by ICC, was classified as poor (< 0.50), moderate (0.50−0.74), good (0.75−0.89), or excellent (≥ 0.90) [32]. The agreement based on kappa coefficients was categorized as poor (< 0.00), slight (0.00−0.20), fair (0.21−0.40), moderate (0.41−0.60), substantial (0.61−0.80), or almost perfect (0.81−1.00) [32]. CT, computed tomography; CI, confidence interval; HU, hounsfield units; ICC, intraclass correlation coefficient.

**Table S2.** Univariable and multivariable analysis for predicting IAC and ILs based on CT indicators in group II

| Variables | *P*-value | | Multivariable analysis (IACs) | | Multivariable analysis (ILs) | |
| --- | --- | --- | --- | --- | --- | --- |
|  | IACs | ILs | *P*-value | OR (95% CI) | *P*-value | OR (95% CI) |
| Attenuation (HU) | 0.113 ^c^ | 0.437 ^c^ |  |  |  |  |
| Diameter (mm) | 0.007 ^c^ | 0.002 ^c^ | 0.791 |  | 0.110 |  |
| Volume (mm^3^) | < 0.001 ^c^ | 0.005 ^c^ |  |  |  |  |
| Mass (mg) | < 0.001 ^c^ | 0.001 ^c^ | 0.031 | 1.004 (1.000–1.008) | 0.592 |  |
| Shape | < 0.001 ^a^ | 0.006 ^a^ | 0.324 |  | 0.389 |  |
| Boundary | 0.495 ^a^ | 0.747 ^b^ |  |  |  |  |
| Lobulation | < 0.001 ^a^ | < 0.001 ^a^ | 0.824 |  | 0.167 |  |
| Spiculation | 0.167 ^b^ | 0.452 ^b^ |  |  |  |  |
| Pleural indentation | 0.602 ^a^ | 0.533 ^b^ |  |  |  |  |
| Vacuole sign | 0.683 ^a^ | 0.330 ^b^ |  |  |  |  |
| Air bronchogram | 0.002 ^a^ | 0.039 ^a^ | 0.263 |  |  |  |

Note: IAC, invasive adenocarcinomas; ILs, invasive lesions; OR, Odds ratio; CI, Confidence interval; HU, hounsfield units

^a^ Calculated by Pearson's chi-square test

^b^ Calculated by Fisher's exact test

^c^ Calculated by Mann-Whitney U test

**Table S3.** Univariable and multivariable analysis for predicting IAC and ILs based on CT indicators in group III

| Variables | *P*-value | | Multivariable analysis (IACs) | | Multivariable analysis (ILs) | |
| --- | --- | --- | --- | --- | --- | --- |
|  | IACs | ILs | *P*-value | OR (95% CI) | *P*-value | OR (95% CI) |
| Attenuation (HU) | 0.266 ^c^ | 0.710 ^c^ |  |  |  |  |
| Diameter (mm) | 0.219 ^c^ | 0.638 ^c^ |  |  |  |  |
| Volume (mm^3^) | 0.032 ^c^ | 0.028 ^c^ |  |  |  |  |
| Mass (mg) | 0.013 ^c^ | 0.022 ^c^ | 0.002 | 1.005 (1.002–1.011) | 0.232 |  |
| Shape | 0.007 ^b^ | 0.011 ^a^ | 0.315 |  | 0.732 |  |
| Boundary | 1.000 ^b^ | 0.508 ^b^ |  |  |  |  |
| Lobulation | 0.004 ^b^ | 0.002 ^a^ | 0.123 |  | 0.065 |  |
| Spiculation | 0.105 ^b^ | 0.056 ^b^ |  |  |  |  |
| Pleural indentation | 0.021 ^b^ | 0.205 ^b^ | 0.246 |  |  |  |
| Vacuole sign | 0.094 ^b^ | 0.741 ^b^ |  |  |  |  |
| Air bronchogram | 0.150 ^b^ | 0.760 ^a^ |  |  |  |  |

Note: IAC, invasive adenocarcinomas; ILs, invasive lesions; OR, Odds ratio; CI, Confidence interval; HU, hounsfield units

^a^ Calculated by Pearson's chi-square test

^b^ Calculated by Fisher's exact test

^c^ Calculated by Mann-Whitney U test

**Figure S1.** Schematic diagram of SSNs size measurement. (a) The size of pGGNs is measured as the average of the longest diameter (blue dashed line) and its perpendicular diameter (pink dashed line) in the same section. (b) For PSNs, nodule size is measured by averaging the longest diameter (blue dashed line) and its perpendicular diameter (pink dashed line) on the same section, covering the entire lesion including both the solid component and peripheral ground-glass component. (c) The measurement of the solid component in PSNs is performed on their largest section by averaging the longest diameter (blue dashed line) and its perpendicular diameter (pink dashed line).

**Figure S2.** Representative CT images and corresponding histopathology of AAH, AIS, MIA, and IAC. (a, b) Axial thin-section CT scan shows a pGGN (arrow) with the pathological diagnosis of AAH, which present as focal atypical alveolar epithelial hyperplasia. (c, d) Axial thin-section CT scan shows a pGGN with the pathological diagnosis of AIS, which present as lepidic growth without stromal, vascular, or pleural invasion. (e, f) Axial thin-section CT scan shows a PSN with a small solid component, pathologically diagnosed as MIA, which predominantly present as lepidic growth with an invasive focus ≤ 0.5 cm. (g, h) Axial thin-section CT scan shows a PSN with the pathological diagnosis of IAC, which present as definitive invasive growth with various histologic patterns.
