# Supplementary figures and images for: Correlation between CT growth patterns and invasiveness progression in neoplastic subcentimeter sub-solid nodules
Source: Ann Med. 2025 Dec 8;57(1):2596471. doi: 10.1080/07853890.2025.2596471 (PMC12687892; doi:10.1080/07853890.2025.2596471)

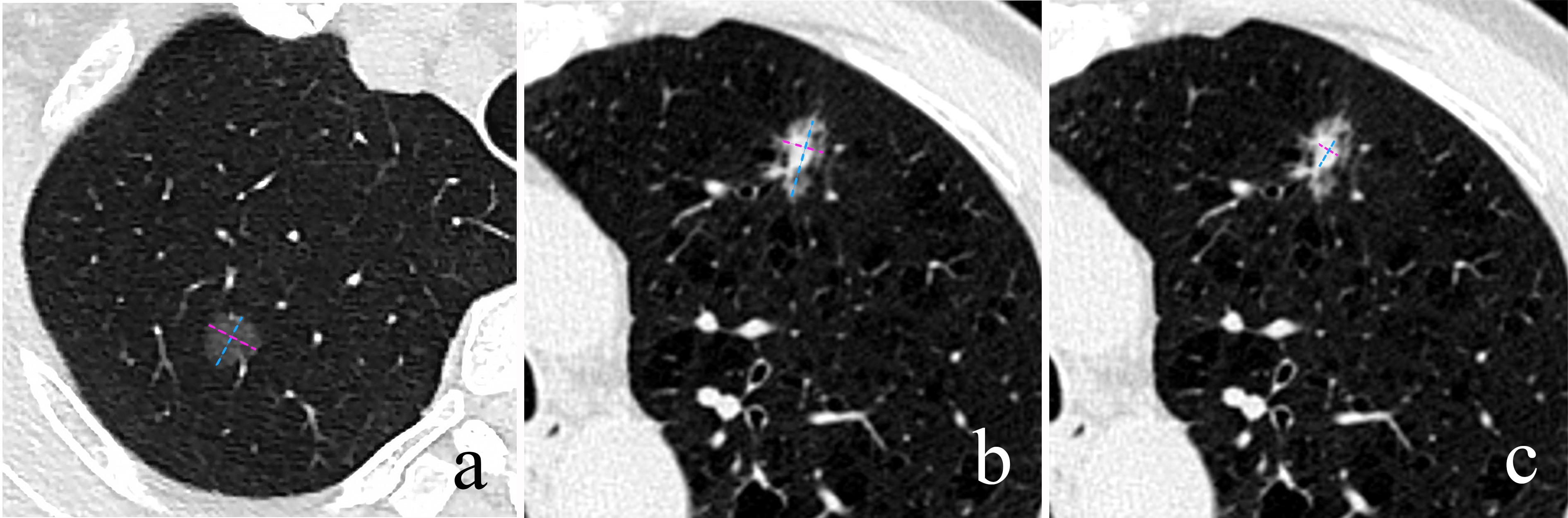

Supplement: Supplemental Material [file IANN_A_2596471_SM0678.zip › suppl_data/Figure S1.tif]

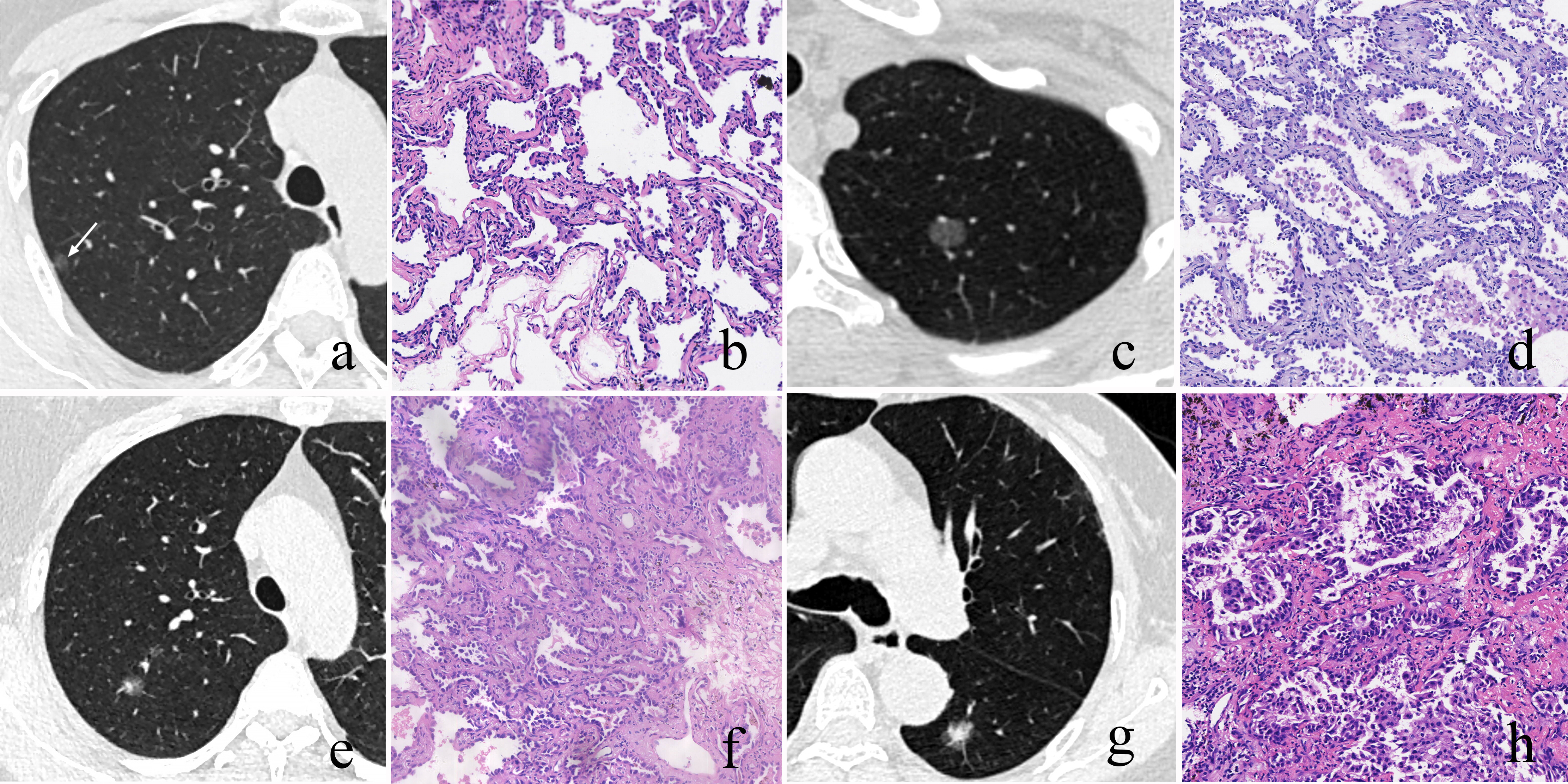

Supplement: Supplemental Material [file IANN_A_2596471_SM0678.zip › suppl_data/Figure S2.tif]
